# Effect of Activating Agent on the Properties of TiO_2_/Activated Carbon Heterostructures for Solar Photocatalytic Degradation of Acetaminophen

**DOI:** 10.3390/ma12030378

**Published:** 2019-01-25

**Authors:** Manuel Peñas-Garzón, Almudena Gómez-Avilés, Jorge Bedia, Juan J. Rodriguez, Carolina Belver

**Affiliations:** Departamento de Ingeniería Química, Facultad de Ciencias, Universidad Autónoma de Madrid, Campus Cantoblanco, E-28049 Madrid, Spain; almudena.gomeza@uam.es (A.G.-A.); jorge.bedia@uam.es (J.B.); juanjo.rodriguez@uam.es (J.J.R.); carolina.belver@uam.es (C.B.)

**Keywords:** Lignin, activating agent, TiO_2_/activated carbon, heterostructures, solar photocatalysis, water purification, acetaminophen

## Abstract

Several activated carbons (ACs) were prepared by chemical activation of lignin with different activating agents (FeCl_3_, ZnCl_2_, H_3_PO_4_ and KOH) and used for synthesizing TiO_2_/activated carbon heterostructures. These heterostructures were obtained by the combination of the activated carbons with a titania precursor using a solvothermal treatment. The synthesized materials were fully characterized (Wavelength-dispersive X-ray fluorescence (WDXRF), X-ray diffraction (XRD), Scanning electron microscopy (SEM), N2 adsorption-desorption, Fourier transform infrared (FTIR) and UV-visible diffuse reflectance spectra (UV-Vis DRS) and further used in the photodegradation of a target pharmaceutical compound (acetaminophen). All heterostructures were composed of anatase phase regardless of the activated carbon used, while the porous texture and surface chemistry depended on the chemical compound used to activate the lignin. Among all heterostructures studied, that obtained by FeCl_3_-activation yielded complete conversion of acetaminophen after 6 h of reaction under solar-simulated irradiation, also showing high conversion after successive cycles. Although the reaction rate was lower than the observed with bare TiO_2_, the heterostructure showed higher settling velocity, thus being considerably easier to recover from the reaction medium.

## 1. Introduction

In recent decades, the treatment of contaminants of emerging concern in water effluents, such as pharmaceuticals and personal care products (PPCPs), is receiving special attention because of their commonly recalcitrant and toxic character [1,2,3]. Those species enter to water bodies through industrial discharges but also from municipal wastewaters [4,5]. In wastewater treatment plants (WWTPs), organic matter and suspended solids can be efficiently removed, but most PPCPs are highly resistant to conventional biological treatments, such as the activated sludge. In this context, there is a growing demand for technologies that can deal with these emerging contaminants in cost-effective terms. Advanced oxidation processes (AOPs), which include Fenton-based, UV- and sunlight-assisted, ozonation or heterogeneous catalytic systems, among others, have been so far widely investigated technologies for that purpose [6,7,8]. Heterogeneous photocatalysis offers the advantage of operating at mild conditions and the opportunity of using solar light as a sustainable and cost-effective energy source, which is a main challenge regarding the economy of this potential solution. In heterogeneous photocatalysis, the irradiation of a semiconductor induces the generation of charges (electrons, e^−^ and holes, h^+^) that, on the surface of the solid, promote the formation of highly oxidizing species, such as hydroxyl (HO^•^) or superoxide (O_2_^•−^) radicals. The energy absorbed by the semiconductor has to be enough to overcome its band gap energy (E_g_), i.e., the energy barrier between the valence and the conduction bands of the material [9]. Titanium dioxide, TiO_2_, is the most used semiconductor among the wide variety of photocatalysts investigated for the treatment of water. It presents the advantages of high photochemical activity and stability, low-toxicity and affordable costs. However, commercial TiO_2_ powder has quite limited adsorption capacity, due to its low surface area, besides its difficult recovery from the reaction medium because of its small particle size, within the nanometric scale [10].

Combining TiO_2_ with carbonaceous materials can be a useful approach to overcome those drawbacks. Carbonaceous materials mainly include activated carbon, carbon fibers or nanotubes and graphene. Among them, activated carbons (ACs) have the lowest cost [11,12]. ACs are amorphous carbonaceous solids with high and well-developed porosity, with surface areas from 500 to 3,000 m^2^·g−^1^, in the whole range of micro- (<2 nm), meso- (2–50 nm) and macropores (>50 nm). Biomass materials, including residues such as agricultural, forestry and associated industrial wastes, can be used as precursors, thus providing valorization opportunities. This is the case of lignin, a high C-content natural biopolymer which is one of the main constituents of lignocellulosic materials, in particular of wood. Cellulose pulp manufacture leaves huge amounts of modified lignin in the black liquors, which is mostly used as fuel. The future biorefinery is expected to produce also high quantities of lignin [13,14,15], which will demand the development of valorization approaches.

The activation of a carbon precursor can be achieved through two main well-known ways: i) physical activation, consisting most commonly in previous pyrolysis of the precursor and subsequent partial gasification of the resulting char with steam or CO_2_; and ii) chemical activation, where the carbon precursor is physically mixed with the activating agent and heat-treated in inert atmosphere [16,17,18,19]. This second method gives in general higher carbon yield due to the combined processes of dehydration and tars inhibition promoted by the activating agent. The activating agents are transition metal salts (e.g., FeCl_3_ or ZnCl_2_), acids (as H_3_PO_4_ or H_2_SO_4_) and alkaline bases (such as KOH or NaOH) [20,21,22,23], playing an important role on the development of different porous textures [24]. The mass ratio between the carbonaceous precursor and the activating agent is also considered a determining variable in that respect [16,18]. The chemical agent also has a very significant effect on the surface chemistry of the resulting activated carbons.

The use of TiO_2_/activated carbon heterostructures for the photocatalytic treatment of PPCPs has been investigated in the last decade. Awfa et al. [11] have recently reported a critical review on this topic. Ultrapure water solutions with high concentrations of pollutants (from 10 to 150 mg·L^−1^) have been tested under simulated sunlight and visible light. Almost complete depletion of the PPCPs was achieved, but in most cases adsorption and photodegradation processes were combined due to the low adsorption times followed in the dark. In this work, different TiO_2_/activated carbon heterostructures were synthesized by solvothermal treatment using a titanium alkoxide as a titania precursor and an activated carbon from chemical activation of lignin with different agents (namely, FeCl_3_, ZnCl_2_, KOH and H_3_PO_4_). The resulting materials were fully characterized and tested in the photodegradation of a pharmaceutical compound (acetaminophen). The novelty of this work is focused on the comparative use of different activating agents to obtain several activated carbons that will be used for preparing TiO_2_/activated carbon heterostructures, with the aim to obtain novel photocatalysts for the degradation of a contaminant of emerging concern. Special attention was paid to achieve adsorption equilibrium prior to the photocatalytic test, evaluating all heterostructures at similar initial concentration of the target compound and equal TiO_2_ content in each experiment under simulated solar light. Finally, the separation of the photocatalyst by settling and its performance and stability after recycles were checked.

## 2. Materials and Methods

### 2.1. Materials

Lignin (from LignoTech Ibérica S.A., Cantabria, Spain) was selected as the carbon precursor for activated carbons. FeCl_3_·6H_2_O (≥97%), ZnCl_2_ (97%), H_3_PO_4_ (85%) and KOH (85%), used as agents for the chemical activation of lignin, were all purchased from Panreac (Panreac Química S.L.U., Barcelona, Spain). Titanium tetrabutoxide (Ti(OBu)_4_; ≥97%) was supplied by Sigma Aldrich (Sigma-Aldrich Co., St. Louis, MO, USA). Ethanol (EtOH; 96%) was obtained from Panreac (Panreac Química S.L.U., Barcelona, Spain). HCl (≥37%) and NaOH (≥95%) were purchased from Sigma Aldrich (Sigma-Aldrich Co., St. Louis, MO, USA) and Scharlau (Scharlab S.L., Barcelona, Spain), respectively. Acetaminophen (ACE; ≥99%, from Sigma-Aldrich Co., St. Louis, MO, USA) was selected as the target emerging contaminant. Acetonitrile (HPLC grade, Scharlau, Scharlab S.L., Barcelona, Spain) and acetic acid (≥99%, Sigma Aldrich Sigma-Aldrich Co., St. Louis, MO, USA) were used as the mobile phase for liquid chromatography. Ultrapure water (Type I, 18.2 MΩ·cm) and deionized water (Type II) were used in this work.

### 2.2. Synthesis of TiO_2_/AC Heterostructures

#### 2.2.1. Preparation of Activated Carbons

Activated carbons were obtained by chemical activation of lignin using different activating agents. Table 1 summarizes the activation conditions of the different carbons [20,25,26,27]. Firstly, 5 g of lignin and the corresponding mass of activating agent were physically mixed. For KOH, the lignin was previously carbonized at 800 °C for 2 h, avoiding the fragmentation and solubilization of the lignin caused by strongly nucleophilic hydroxyl ions from the activating agent [28]. Then, the mixtures were dried at 60 °C overnight and heat-treated at the desired temperature for 2 h under N_2_ flow (100 Ncm^3^·min^−1^) in a horizontal stainless-steel tube furnace, using a heating rate of 10 °C ·min^−1^. Then, the samples were cooled down to room temperature under N_2_ flow. The resulting solids were further washed in two steps. Firstly, with HCl (0.1 M) at 70 °C for 2 h to remove the residual activating agent and secondly, with deionized water at room temperature up to neutral pH. The final materials were dried overnight in an oven at 60 °C. The resulting activated carbons were denoted as Fe-C, Zn-C, P-C and K-C, according to the activating agent used. The activating agent to lignin mass ratio was established in each case after previous experiments where different values were tested.

#### 2.2.2. Solvothermal Synthesis of TiO_2_/Activated Carbon Heterostructures

TiO_2_/activated carbon heterostructures were obtained following a solvothermal synthesis [29]. Preliminary trials were conducted where different TiO_2_/AC mass ratios were checked prior to select the most suitable to achieve heterostructures based on anatase phase. After these studies, the amount of TiO_2_ was fixed at 80% in all cases. Thus, 58 mg of activated carbon were suspended into 45 mL of EtOH at room temperature for 5 min, leading to the solution A. At the same time, 1 mL of Ti(OBu)_4_ was diluted in 15 mL of EtOH for 5 min (solution B). Then, solution B was added dropwise to solution A under continuous stirring until complete homogenization. A solution of 3 mL of ultrapure water in 15 mL of EtOH was incorporated dropwise to produce the hydrolysis of the Ti precursor. The mixture was stirred for 5 min, transferred to a 125 mL Teflon-lined stainless-steel autoclave, and heated at 160 °C for 3 h. After the reaction, the solid was separated by centrifugation (5300 rpm, 10 min), washed three times with deionized water and finally with ethanol. The resulting grey materials were dried at 60 °C overnight. The heterostructures were labelled as TiO_2_/x-C, namely TiO_2_/Fe-C, TiO_2_/Zn-C, TiO_2_/P-C and TiO_2_/K-C, depending on the activated carbon used to form the heterostructure. For comparison, bare TiO_2_ was also obtained under the same conditions in the absence of activated carbon. 

### 2.3. Characterization Techniques

A Bruker S8 TIGER spectrometer (Bruker, Billerica, MA, USA) under inert atmosphere (He) (maximum voltage of 60 kV and maximum current of 170 mA) was used to determine the percentage of TiO_2_ in the final samples by wavelength-dispersive X-ray fluorescence (WDXRF). X-ray diffraction (XRD) patterns were recorded on a Bruker D8 diffractometer (Bruker, Billerica, MA, USA) with a scintillation detector, using Cu-Kα source, with a scan step of 1°∙min^−1^ between 5 and 70° of 2θ. Scherrer’s equation was used to estimate the average crystal size (D) from the most intense diffraction peak (101) of anatase phase. A Quanta 3D Field Emission Gun (FEG) microscope (FEI Company, Hillsboro, OR, USA) was used to obtain the scanning electron microscopy (SEM) images of the samples. The particles size distributions were obtained from these images by using the ImageJ software (National Institutes of Health, Bethesda, MD, USA). In each case, 20 particles per image (out of a total of five images) were analyzed to achieve a good representation of the particle size.

The porous texture was characterized by N_2_ adsorption-desorption at −196 °C using a Micromeritics TriStar 123 static volumetric system (Micromeritics Instrument Corp., Norcross, GA, USA). The samples were previously outgassed under vacuum at 150 °C overnight in a Florprep 060 Micromeritics device (Micromeritics Instrument Corp., Norcross, GA, USA). The specific surface area (S_BET_) was determined by the Brunauer-Emmett-Teller (BET) method [30], while the external or non-microporous surface area (S_EXT_) and micropore surface area (S_MP_) were calculated using the t-plot method [31]. Fourier Transform Infrared (FTIR) spectra (wavenumber range 4000–400 cm^−1^) were recorded on a Bruker iFS 66VS spectrometer (Bruker, Billerica, MA, USA) using a resolution of 2 cm^−1^. Samples were previously prepared using KBr pellets. The pH drift method [32] was used to determine the pH at the point of zero charge (pH_pzc_). Briefly, 50 cm^3^ of 0.01 M NaCl solution at initial pH (adjusted between 3–11 using 0.1 M HCl or NaOH) were placed in a closed titration vessel. Then, 20 mg of the sample were suspended and nitrogen was bubbled before starting the test in order to stabilize the initial pH by removing dissolved gasses. The final pH, measured after 5 h, was plotted versus the initial one. The pH_pzc_ is given by value where the curve crosses pH_initial_ = pH_final_.

A Shimadzu 2501PC UV-vis spectrophotometer (Shimadzu Corporation, Kyoto, Japan) was used to record UV-vis diffuse reflectance spectra (UV-vis DRS) in the 250–800 nm region using BaSO_4_ as reference material. The band gap values were estimated using the Tauc Plot standard technique [33]. Considering that all heterostructures are indirect semiconductors, as TiO_2_ [34], this method uses the equation αhν = α·(hν−E_g_)^1/2^, where α, h, ν and E_g_ are the absorption coefficient, Planck constant, light frequency and the energy gap of the semiconductor, respectively. Plotting (αhυ)^1/2^ vs. hυ results in a curve with a linear region. The extrapolation of this linear branch to the X-axis provides the band gap value of the material.

### 2.4. Photocatalytic Tests

The photocatalytic degradation of acetaminophen (ACE) was performed in a 500 mL Pyrex jacketed reactor (Segainvex UAM, Madrid, Spain) at a controlled temperature of 25 °C. The experiments were carried out inside a Suntest solar simulator (Suntest XLS+, ATLAS, Mount Prospect, IL, USA) equipped with a 765–250 W·m^−2^ Xe lamp. Solar radiation was simulated using a “Daylight” filter (cuts off λ ≤ 290 nm), selecting 600 W·m^−2^ (107.14 klx) as irradiation intensity. In each test, the concentration of photocatalyst was set so that all the experiments were performed with the same amount of TiO_2_, 250 mg·L^−1^. For this purpose, the results obtained after characterization by WDXRF were used to know the amount of TiO_2_ of each sample. The calculated amount of photocatalyst was dispersed in 150 mL deionized aqueous solution containing the contaminant. Prior to the photocatalytic tests, the adsorption capacity of each heterostructure was estimated since the heterostructures showed very different porous textures. Hereby, each catalyst was contacted with ACE solutions of different concentrations, measuring the amount adsorbed at equilibrium after 16 h. That value was used to adjust the initial concentration of ACE for each catalyst, which was in all cases 5 mg·L^−1^. Further, the suspension was exposed to simulated solar light for 6 h. Samples of 450 µL were collected at different times and filtered using PTFE syringeless filters (Scharlau, Scharlab S.L., Barcelona, Spain) (Whatman 0.2 µm). The liquid phase was analyzed by HPLC (Shimadzu Prominence-I LC-2030C, Shimadzu Corporation, Kyoto, Japan) equipped with a diode array detector (SPD-M30A) and a reverse phase C18 column (Eclipse Plus 5 µm, Agilent Technologies, Santa Clara, CA, USA) to measure the ACE concentration (detection wavelength set at 246 nm). A mixture of acetonitrile/acetic acid 0.1% *v/v* (gradient method: 10/90–40/60% (0–17 min)) was used as the mobile phase, with a constant flow of 0.7 mL·min^−1^. Total organic carbon (TOC) was measured at the beginning and end of the reaction using a Shimadzu TOC-L analyzer (Shimadzu Corporation, Kyoto, Japan). The experiments were carried out in duplicate. Settling tests were performed with catalysts suspensions of 1 g·L^−1^. The settling profiles versus time were recorded using a Shimadzu 2501PC UV-vis spectrophotometer (Shimadzu Corporation, Kyoto, Japan) (double beam), measuring the absorbance at 600 nm (in which the extinction of light is mainly due to the scattering caused by the particles of the suspension). In these tests, 4 mL of the suspension were placed in a quartz cuvette and the absorbance were recorded continuously for 2 h. Deionized water in absence of the catalyst was used as blank. In parallel, 100 mL of the catalysts suspensions with the same concentration were placed in graduated cylinders allowing the natural sedimentation of the particles. Pictures at different time intervals were taken for comparison.

## 3. Results and Discussion

### 3.1. Characterization of TiO_2_/Activated Carbon Heterostructures

**Figure 1:**
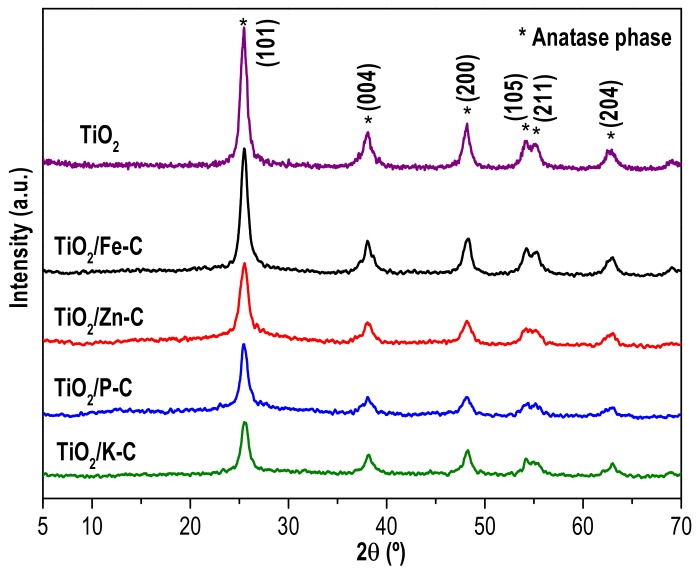
XRD patterns of the TiO_2_/activated carbon heterostructures and bare TiO_2_. Characteristic peaks of anatase phase (JCPDS-78–2486) are indicated (*).

The XRD diffractograms of the TiO_2_/x-C heterostructures synthesized are depicted in Figure 1 together with that of the TiO_2_ prepared as reference. All the samples show the characteristic peaks of the anatase phase (JCPDS file No. 78-2486), whose (hkl) planes are marked in the Figure 1. No peaks assigned to other crystal phases of TiO_2_ (e.g., rutile and brookite) or other from the AC were detected. That was also the situation with TiO_2_/C heterostructures synthesized by a similar procedure using glucose as carbon precursor [29]. Thus, solvothermal synthesis at 160 °C allowed the development of the crystalline structure of titania without the need of further heat treatment. Comparing the diffractograms, the most intense peak of anatase (101) differs in width depending of the samples. Usually, the widening of the diffraction peaks is associated with the reduction of the crystal size and the creation of defects in the crystalline structure. The anatase crystal size (D) was calculated by using the Scherrer’s equation and the position and width of the (101) peak. The values are collected in Table 2. The bare TiO_2_ prepared by this solvothermal treatment exhibits a small crystal size, lower than that reported for anatase TiO_2_ prepared by other methods [35]. In the TiO_2_/x-C samples, even smaller TiO_2_ crystal sizes are observed, the values depending on the nature of the activating agent used to prepare the AC. The lowest anatase crystal size was obtained with Zn-C. These values are analogous to those reported for other TiO_2_/AC hybrids prepared by the sol-gel method that required a calcination step in air [36,37]. The amount of TiO_2_ incorporated in the heterostructures was determined by WDXRF and the results are included in Table 2. Most of the samples have a TiO_2_ content similar to the expected (80%), with only the sample TiO_2_/Zn-C showing some significant deviation. Additional samples were prepared by decreasing the percentage of TiO_2_, but the results obtained in terms of photocatalytic activity for ACE degradation were considerably poorer and thus, those samples were discarded.

**Figure 2:**
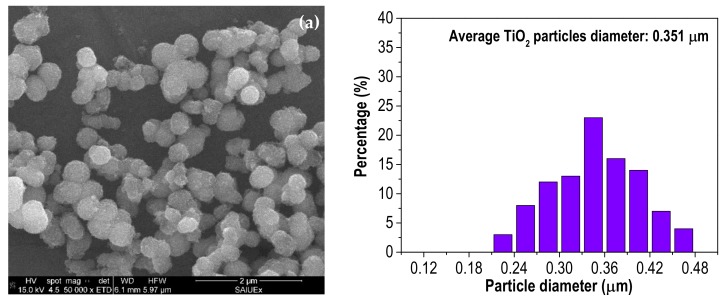

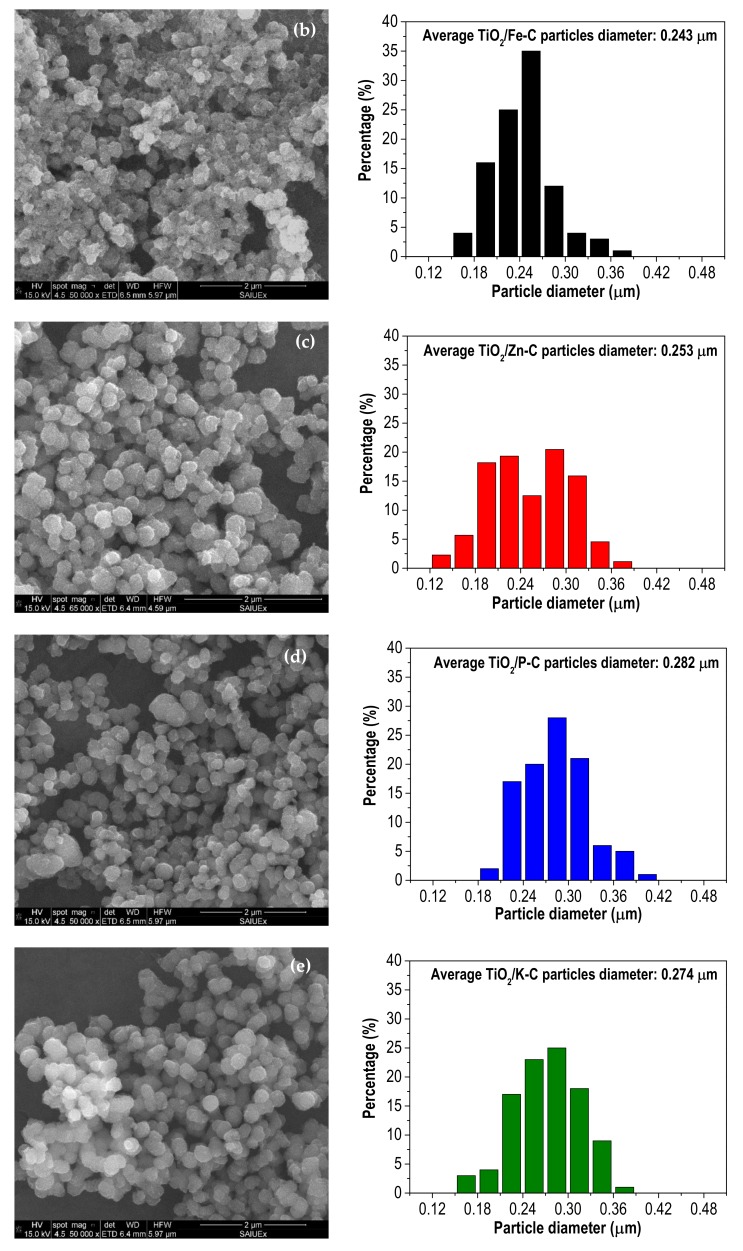
SEM images and particle size distribution of: (**a**) TiO_2_; (**b**) TiO_2_/Fe-C; (**c**) TiO_2_/Zn-C; (**d**) TiO_2_/P-C; (**e**) TiO_2_/K-C.

All synthesized heterostructures showed an external spherical-like morphology, as it can be observed from the SEM images (Figure 2), analogous to the bare TiO_2_. Figure 2 also includes the histograms of the particle size distribution, obtained by measuring ca. to 100 particles for each sample. The average diameter of TiO_2_ particles is higher than the obtained for the heterostructures, which fall within the range of 0.24–0.28 μm. However, the particles of TiO_2_/x-C show a higher degree of agglomeration. This suggests that the presence of activated carbon inhibits the growth of the TiO_2_ particles, as previously reported by Wang et al. [38]. The size distribution is unimodal in all cases except for TiO_2_/Zn-C, which shows a bimodal profile centered at 0.21 and 0.30 μm.

**Figure 3:**
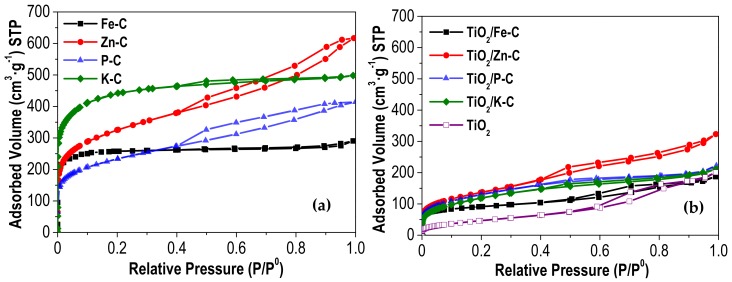
N_2_ adsorption-desorption isotherms (−196 °C) of (**a**) activated carbons and (**b**) TiO_2_ and TiO_2_/x-C heterostructures.

Nitrogen adsorption–desorption isotherms of activated carbons and TiO_2_/x-C heterostructures are represented in Figure 3. K-C and Fe-C ACs (Figure 3a) show typical type I isotherms, characteristic of microporous materials, according to the IUPAC classification [39]. In both cases, the major uptake occurs at low relative pressures followed by an almost horizontal branch, this shape being indicative of highly microporous solids. In contrast, Zn-C and P-C describe hybrid I/II type isotherms with hysteresis loops at P/P_0_ > 0.4, suggesting the existence of a well-developed microporous structure but with significant contribution of mesoporosity. Figure 3b shows that the different TiO_2_/x-C heterostructures present type II isotherms, with a remarkable decrease of adsorption, indicating that the porous structure of the activated carbons has been partially blocked by the incorporation of TiO_2_. 

Table 3 summarizes the surface area values (BET, microporous and external or non-microporous) characterizing the porous texture of the synthesized materials. The development of surface area depends on the chemical activating agent used, following the order K-C >> Zn-C >> P-C > Fe-C. It should be mentioned the high development of mesoporosity upon ZnCl_2_-activation (as showed by the high value of the so-called external surface area). According to literature [28,40,41], the chemical activation mechanisms consist in the oxidation and/or dehydration of the precursor, including complex different reactions depending on the agent. However, a general mechanism involved in this type of activation is still not very clear. The dehydration during the heat treatment of the lignin seems to be the most determinant effect in the chemical activation with ZnCl_2_ and H_3_PO_4_. In the case of H_3_PO_4_, a strong Brønsted acid, it yields a partial depolymerization, followed by dehydration and condensation processes; whereas in the case of ZnCl_2_, a Lewis acid, the reaction with lignin is proton-catalyzed producing the dehydration and further aromatization of the carbon skeleton [42]. KOH reacts with the solid precursor by means of redox reactions, resulting in the microporosity development after oxidizing carbon to CO and CO_2_. This predominant formation of micropores has also been observed with FeCl_3_-activation, in contrast to ZnCl_2_, ascribing a similar behavior to that of alkali agents [20]. In the case of TiO_2_, the entire surface area corresponds to mesopores. Further introduction of activated carbon in the solvothermal synthesis step allowed obtaining heterostructures including microporosity.

**Figure 4:**
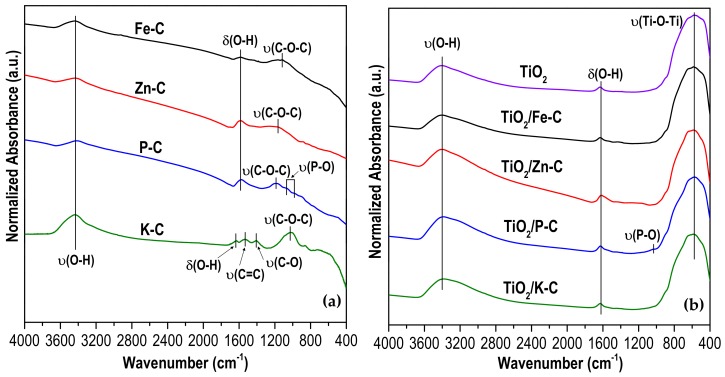
FTIR spectra of: (**a**) activated carbons; (**b**) TiO_2_ and TiO_2_/x-C heterostructures.

The surface functional groups of all activated carbons and synthesized heterostructures were assessed from the FTIR spectra (Figure 4). The activated carbons (Figure 4a) show the characteristic bands due to the presence of adsorbed water, appearing the –OH stretching and bending bands at 3400 and 1600 cm^−1^, respectively [29,43,44]. In the K-C carbon, the peaks located at 1537 and 1408 cm^−1^ were assigned to the stretching of –C=C in the skeletal aromatic ring and –CO stretching of carbonate groups, respectively [43,45,46]. Stretching of –COC can be observed in the absorption bands centered in the range of 1170–1030 cm^−1^. This vibration can be produced from diverse oxygenated groups, thus varying the main absorption peak [29,43]. In P-C, the weak bands centered at 1060 and 980 cm^−1^ can be attributed to –PO and –POC groups. TiO_2_ and TiO_2_/x-C FTIR spectra (Figure 4b), are very similar among them, indicating the homogeneous distribution of TiO_2_ over the heterostructure. There are three main absorption bands. Those centered at 3400 and 1625 cm^−1^ are associated with adsorbed water, as previously indicated for activated carbons. The wide band located between 800 and 400 cm^−1^ corresponds to the characteristic –Ti-O-Ti stretching band of TiO_2_ [47,48]. This band appears in all samples, with a maximum ca. to 700 cm^−1^, which corroborates the generation of titania phase in all the heterostructures. The high intensity and width of this band overlaps those characteristic bands described for the activated carbons, taking into account that the TiO_2_ percentage of these heterostructures is fairly high (ca. 80%). In the case of TiO_2_/P–C, around 1030 cm^−1^ appears a weak shoulder that can be ascribed to the stretching bands of –PO and –POC groups previously described for the corresponding activated carbon.

An important parameter regarding the acid-base behavior of the samples is the pH_pzc_. When the pH of the solution is lower than the pH_pzc_, the surface of the solid is positively charged, whereas it is negatively charged at pH above the pH_pzc_ of the sample. Table 2 summarizes the pH_pzc_ values of the synthesized heterostructures. The pH_pzc_ of bare TiO_2_ is almost neutral, which means that its surface is not charged in neutral water [49,50]. In the case of the heterostructures, it is clear that the pH_pzc_ values depend on the activating agent used during the preparation of the activated carbon, corresponding the lowest value to TiO_2_/P-C. The low acidity of FeCl_3_ (a weak Lewis acid) can result in the introduction of poor acidic groups in the heterostructure surface, giving a pH_pzc_ analogous to that of TiO_2_, while the use of H_3_PO_4_ (a strong Brønsted acid) yields a heterostructure with a low pH_pzc_ probably due to the presence of phosphates that leave OH groups on the surface [17]. Regarding to TiO_2_/K-C, despite the basic character of KOH, the solid has a relatively neutral PZC (even lower than that prepared with FeCl_3_). This may be due to the fact that after activation the solid was washed with HCl to remove the remaining KOH, thus eliminating the basic sites in the final solid.

**Figure 5:**
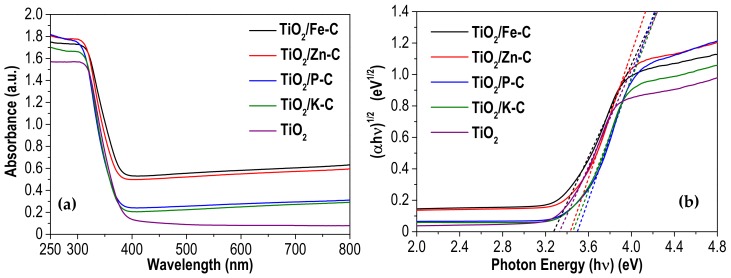
(**a**) UV–vis diffuse absorbance spectra and (**b**) the (αhυ)^1/2^ versus (hυ) plot of the synthesized photocatalysts.

The light absorption in the UV and visible region of the synthesized photocatalysts was investigated by UV-vis DRS technique, the resulting spectra are shown in Figure 5a. The absorption band observed in the UV range (below 360 nm) is very similar in all cases, typical of TiO_2_. In the visible region, the spectra of the heterostructures do not fall to zero because of their grey color. The effect is more evident in TiO_2_/Fe-C and TiO_2_/Zn-C, because these samples have lower percentages of TiO_2_ (Table 2), yielding the color change from light-to-dark grey. As mentioned above, the band gap values were determined from the UV-vis DRS spectra (according to the Tauc plot method, Figure 5b) and are included in Table 2. The values are fairly similar to that of TiO_2_ [51,52], only somewhat higher except in the case of TiO_2_/Fe-C. Although it has been reported that the combination of TiO_2_ with carbonaceous supports can produce a significant red-shifted displacement of absorption edge, in our heterostructures this has not been observed. This may be due to a more limited interaction between TiO_2_ and AC compared to other carbon supports, like graphene or carbon nanotubes [11,12].

### 3.2. Photocatalytic Tests

**Figure 6:**
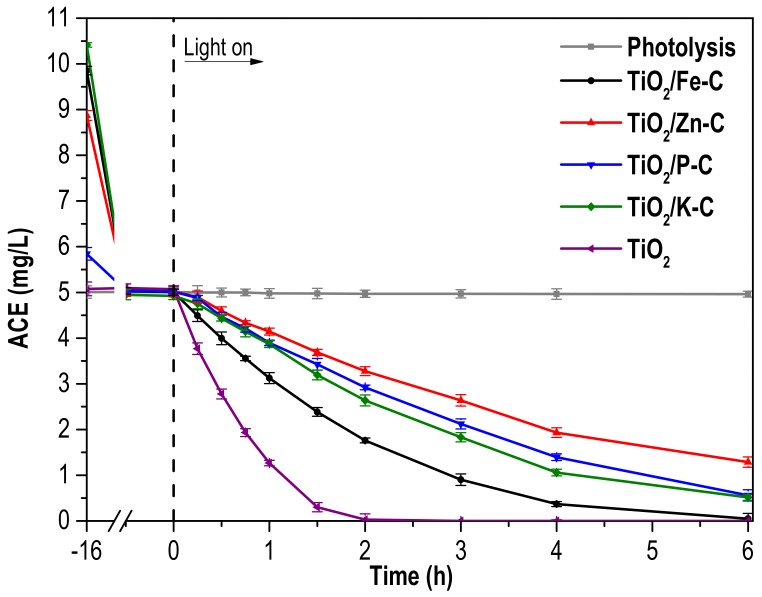
Acetaminophen (ACE) concentration versus time under solar irradiation with TiO_2_ and TiO_2_/x-C heterostructures ([Photocatalyst]_0_: 250 mg·L^−1^ of TiO_2_; [ACE]_0_ after adsorption equilibrium: 5 mg·L^−1^; intensity of irradiation: 600 W·m^−2^).

The photocatalytic performance of the synthesized heterostructures in the degradation of acetaminophen (ACE) under solar light is depicted in Figure 6, which includes the results with bare TiO_2_ for the sake of comparison, as well as a blank experiment, showing the complete stability of ACE under solar light irradiation in absence of catalyst. A comparative study using TiO_2_/Fe-C as photocatalyst was also carried out for 6 h with and without simulated solar radiation (Figure S1) after the adsorption period, showing no variation in the ACE concentration in absence of light. As depicted in Figure 6, the light-assisted tests were preceded by a 16-h step in dark to allow the adsorption equilibrium. The differences in the adsorbed quantity of ACE can be explained by means of multiple interactions, such as electrostatic forces or the porous texture. Negligible ACE is adsorbed on the TiO_2_ surface, probably due to its low porous development. In contrast, the interaction between the heterostructures and the contaminant seems to be more influenced by electrostatic interactions. The reaction was carried out at an initial pH of 6.9. Due to that the pKa of ACE is 9.9, the molecules of the contaminant are neutrally charged [53]. In contrast, pH_pzc_ of the different heterostructures is below than the reaction pH and, thus, the surface of these photocatalysts is partially negatively charged (increasing the negative charge with decreasing the pH_pzc_). As a consequence, the quantity of adsorbed ACE decreases as pH_pzc_ diminishes because of partial repulsive electrostatic forces between the neutral molecule and the negative character of the surface of the heterostructures. For example, TiO_2_/P-C has the lower pH_pzc_ value of synthesized heterostructures (4.86) and show the lowest adsorption of ACE.

It can also be seen in Figure 6 that bare TiO_2_ exhibits better photocatalytic performance than the synthesized TiO_2_/x-C heterostructures, which is consistent with the easier accessibility of TiO_2_ [11], but also with the higher opacity of the suspension caused by the black-grey color. Furthermore, the higher concentration of the heterostructures contributes to their poorer behavior (all the tests were performed with the same amount of TiO_2_, and therefore, the amount of solid in the suspension is higher with the TiO_2_/x-C heterostructures). Regarding the photocatalytic performance of the synthesized heterostructures, TiO_2_/Fe-C appears as the most active, allowing complete ACE conversion after 6 h of reaction, probably due to the lowest band gap of this photocatalyst. The reduction of the photocatalytic activity of TiO_2_/activated carbon heterostructures with respect to bare TiO_2_ has been previously reported [54,55]. However, other publications claimed the opposite behavior, where the highest photocatalytic activity was found for TiO_2_/activated carbon materials [29,56,57]. In some of these works, dark-adsorption was extended for no more than 1 h, which was not probably enough for the complete adsorption equilibrium. Therefore, when the photocatalytic tests started, a combination between adsorption and photocatalytic reaction was carried out. In this sense, the current work focuses only in the photocatalytic performance of the synthesized heterostructures, after reaching adsorption equilibrium. 

Since the degradation pathway is of high interest, the final solution was evaluated by ion-exchange chromatography, founding different small carboxylic acids (acetic, formic and malonic) although their total concentration was really low, below 0.07 mg/L. Following the degradation pathway described in the literature [58,59], the presence of other proposed aromatic compounds was investigated but no one could be elucidated. Mineralization of the target pollutant, i.e., complete conversion into CO_2_ and H_2_O, was followed by measuring the total organic carbon (TOC) in the solution after the 6 h-irradiation time (Table 4). The TiO_2_/x-C heterostructures yielded significantly lower degradation of TOC than the bare TiO_2_, being again TiO_2_/Fe-C the most effective among them.

**Figure 7:**
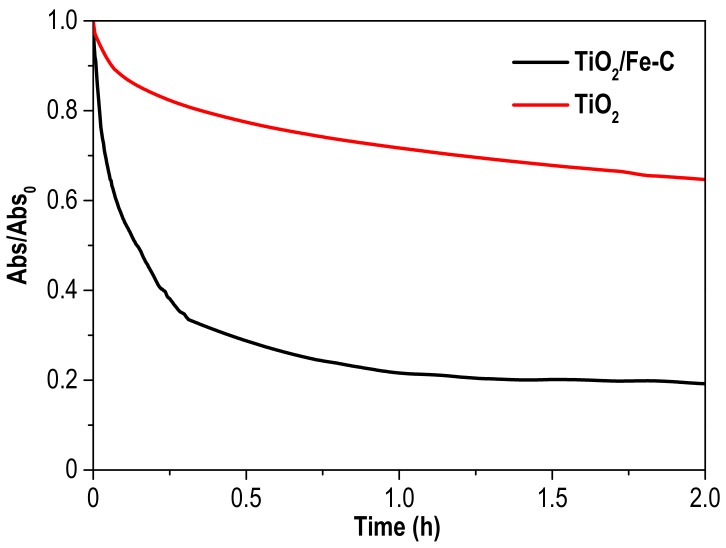
Absorbance evolution profiles (600 nm) during settling test of TiO_2_/Fe-C and TiO_2_ photocatalysts.

The potential application of a photocatalyst must consider not only its activity but also the recovery from the reaction medium. Settling tests were conducted at neutral pH with bare TiO_2_ and TiO_2_/Fe-C and the results demonstrated the significantly easier separation of this last (as depicted in Figure 7), probably due to the higher degree of agglomeration observed by SEM. In addition to this, Figure S2 shows pictures of the settling process for both photocatalysts at different times, in which the highest settling velocity of the TiO_2_/Fe-C can also be observed, thus being considerably easier to recover the heterostructure from the medium.

**Figure 8:**
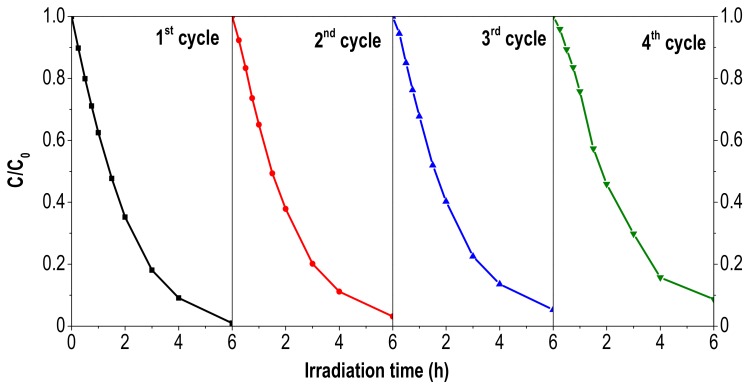
ACE removal with TiO_2_/Fe-C during consecutive cycles (Reaction conditions identical than those from Figure 6).

**Figure 9:**
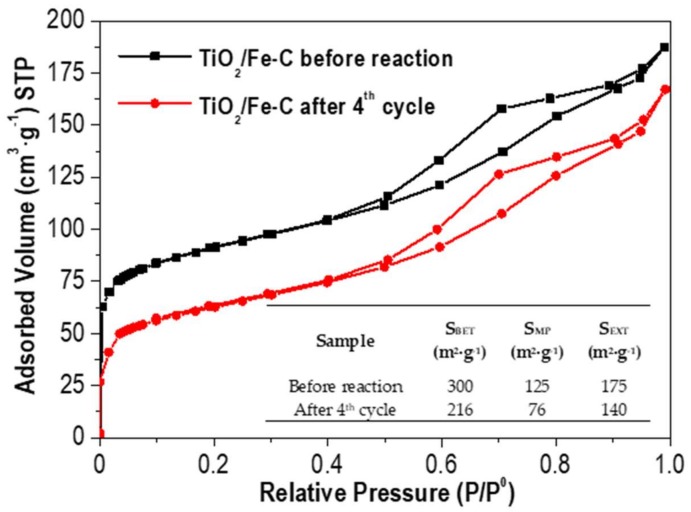
N_2_ adsorption-desorption isotherms (−196 °C) of TiO_2_/Fe-C before reaction and after four consecutive cycles. Surface area values of the photocatalyst are also included in the embedded table.

The stability of the photocatalyst with the best recoverability was investigated upon four consecutive cycles. After each cycle, the used TiO_2_/Fe-C was filtered, washed with deionized water and dried at 60 °C overnight. Each new cycle was carried out with identical conditions as previously described for the degradation of ACE (Figure 6). As depicted in Figure 8, the heterostructure showed a good performance in the photocatalytic oxidation of ACE after four successive cycles with a slight decrease in the final conversion (92% removal after 6 h of irradiation). The porous texture of the used photocatalyst was also analysed (Figure 9), showing a decrease of the porous network, probably due to the partial blocking of the microporous structure by the adsorbed contaminant or even the oxidized intermediates. Furthermore, the leaching of titania was not detected in the solution after four recycles by using inductive coupled plasma methodology. 

## 4. Conclusions

Solvothermal synthesis of TiO_2_/activated carbon (TiO_2_/AC) heterostructures was successfully achieved. Lignin has been used as starting material for the ACs following chemical activation with four agents (FeCl_3_, ZnCl_2_, H_3_PO_4_ and KOH). The activated carbons showed a well-developed porous texture and different surface functional oxygenated groups and acid-basic character depending of the activation procedure. XRD patterns of the TiO_2_/AC heterostructures confirmed the presence of anatase phase with crystal size close to 10 nm in all cases after the solvothermal synthesis, without the need for further heat-treatment. These materials showed a spherical morphology with a particle size close to 0.27 µm. The presence of activated carbon in the heterostructures increased somewhat the band gap with respect to bare TiO_2_, except for TiO_2_/Fe-C. TiO_2_ and TiO_2_/Fe-C showed the best efficiency in the degradation of acetaminophen under solar light, being higher in the case of TiO_2_. However, settling experiments demonstrated the easier recovery of the heterostructured material in spite of the lower size of individual particles, because of the higher aggregation observed by SEM. TiO_2_/Fe-C also showed a good performance in the photocatalytic oxidation of ACE after four successive cycles.

## Figures and Tables

**Table 1:** Activation conditions of the different carbons.

Sample	Activating Agent	Act. Agent: Lignin Mass Ratio	Activation Temperature (°C)
Fe-C	FeCl_3_	3:1	800
Zn-C	ZnCl_2_	3:1	500
P-C	H_3_PO_4_	3:1	500
K-C	KOH	4:1	900

**Table 2:** TiO_2_ content, average crystal size (D), band gap (E_g_) and pH_pzc_ of the heterostructures.

Sample	%TiO_2_^a^	D (nm)^b^	E_g_ (eV)	pH_pzc_^c^
TiO_2_/Fe-C	75.9	10.1	3.28	6.36
TiO_2_/Zn-C	68.5	8.6	3.42	5.96
TiO_2_/P-C	81.3	10.6	3.50	4.86
TiO_2_/K-C	83.9	9.4	3.45	6.17
TiO_2_	n.m	10.5	3.33	6.58

^a^ Determined by WDXRF (n.m. not measured). ^b^ Average crystal size from (101) diffraction peak. ^c^ Determined from the drift method.

**Table 3:** Surface area values of the synthesized materials.

Sample	S_BET_ (m^2^·g^−1^)	S_MP_ (m^2^·g^−1^)	S_EXT_ (m^2^·g^−1^)
Carbonized lignin	62	62	-
Fe-C	756	695	61
Zn-C	1129	451	678
P-C	807	303	504
K-C	1446	1142	304
TiO_2_/Fe-C	300	125	175
TiO_2_/Zn-C	491	108	383
TiO_2_/P-C	435	109	326
TiO_2_/K-C	465	156	309
TiO_2_	178	-	178

**Table 4:** TOC removal after 6 h of solar irradiation with the photocatalysts tested.

Photocatalyst	Removed TOC (%)
TiO_2_/Fe-C	43.3
TiO_2_/Zn-C	23.9
TiO_2_/P-C	35.5
TiO_2_/K-C	30.2
TiO_2_	59.4

## Supplementary Materials

The following are available online at http://www.mdpi.com/1996-1944/12/3/378/s1, Figure S2: Evolution of ACE concentration with and without irradiation using TiO_2_/Fe-C as photocatalyst ([Photocatalyst]_0_: 250 mg·L^−1^ of TiO_2_; [ACE]_0_ after adsorption equilibrium: 5 mg·L^−1^; intensity of irradiation: 600 W·m^−2^); Figure S1: Settling test for TiO_2_/Fe-C (left) and TiO_2_ (right) samples.
