# Supplementary material for: Effect of Activating Agent on the Properties of TiO2/Activated Carbon Heterostructures for Solar Photocatalytic Degradation of Acetaminophen
Source: Materials (Basel). 2019 Jan 25;12(3):378. doi: 10.3390/ma12030378 (PMC6384744; doi:10.3390/ma12030378)
Supplement: Supplementary file 1 [file materials-12-00378-s001.pdf]

Supplementary

Effect of Activating Agent on the Properties of TiO_2/Activated Carbon Heterostructures for Solar Photocatalytic Degradation of Acetaminophen

Manuel Peñas-Garzón, Almudena Gómez-Avilés, Jorge Bedia, Juan J. Rodriguez and Carolina Belver

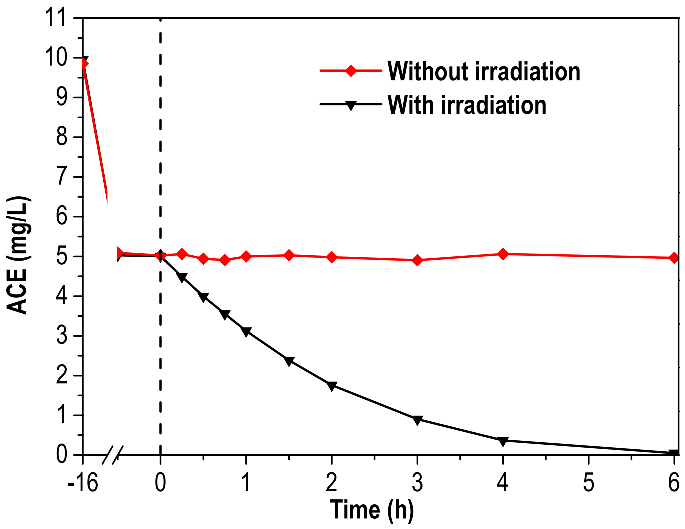

Figure S1. Evolution of ACE concentration with and without irradiation using TiO_2/Fe-C as photocatalyst ($[Photocatalyst]_0$: 250 mg·L^{-1} of TiO_2; $[ACE]_0$ after adsorption equilibrium: 5 mg·L^{-1}; intensity of irradiation: 600 W·m^{-2}).

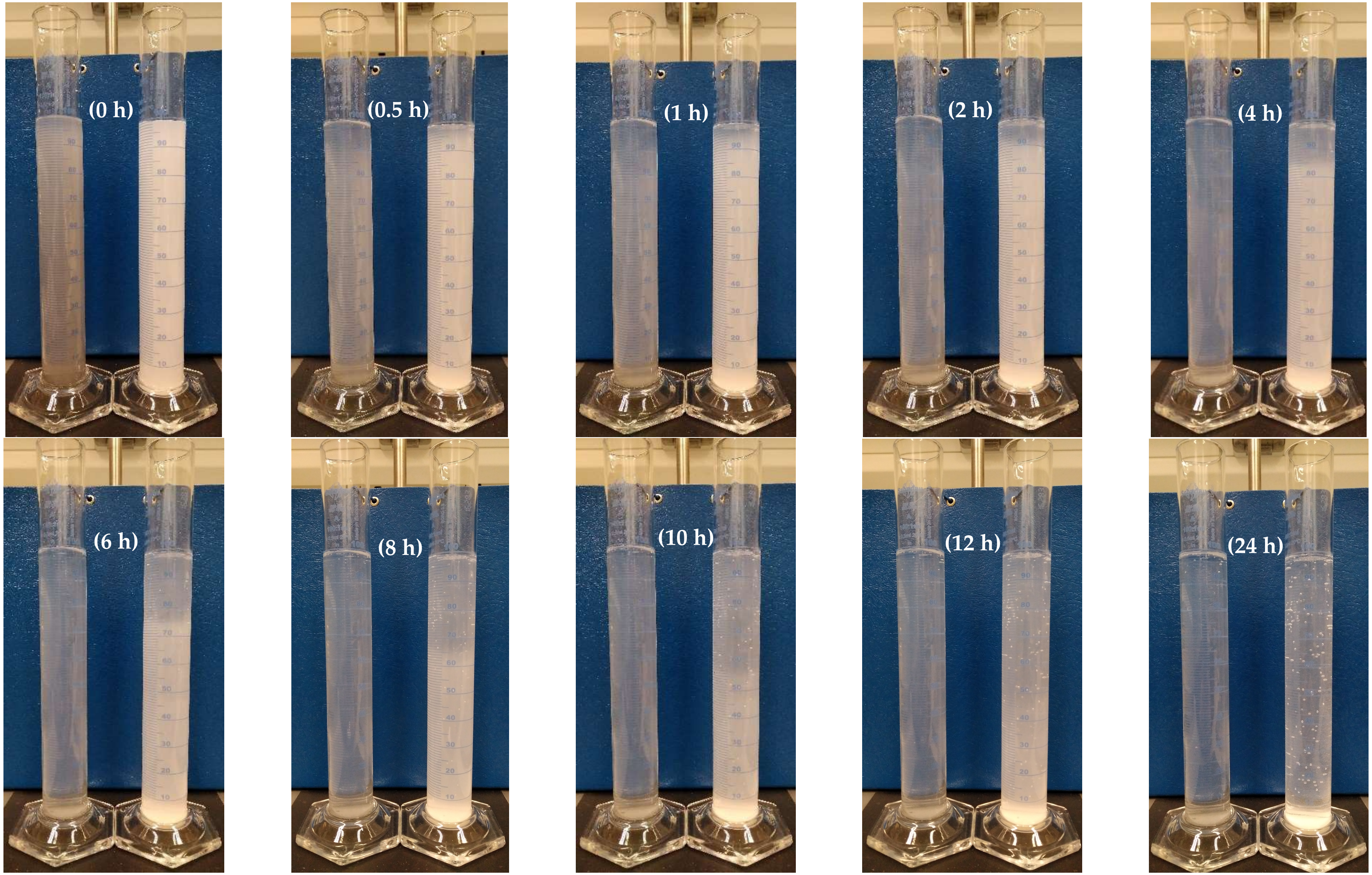

Figure S2. Settling test for TiO_2/Fe-C (left) and TiO_2 (right) samples
